# A machine learning-based approach to predicting the malignant and metastasis of thyroid cancer

**DOI:** 10.3389/fonc.2022.938292

**Published:** 2022-12-19

**Authors:** Jianhua Gu, Rongli Xie, Yanna Zhao, Zhifeng Zhao, Dan Xu, Min Ding, Tingyu Lin, Wenjuan Xu, Zihuai Nie, Enjun Miao, Dan Tan, Sibo Zhu, Dongjie Shen, Jian Fei

**Affiliations:** ^1^ Department of General Surgery, Shanghai Punan Hospital of Pudong New District, Shanghai, China; ^2^ Department of General Surgery, Shanghai Ruijin Rehabilitation Hospital, Shanghai, China; ^3^ Department of General Surgery, Ruijin Hospital Lu Wan Branch, Shanghai Jiaotong University School of Medicine, Shanghai, China; ^4^ Department of Ultrasound, Tongji Hospital, School of Medicine, Tongji University, Shanghai, China; ^5^ Department of General Surgery, Ruijin Hospital, Shanghai Jiao Tong University School of Medicine, Shanghai, China; ^6^ School of Life Sciences, Fudan University, Shanghai, China

**Keywords:** machine learning, thyroid cancer, thyroid nodule, risk prediction, metastasis

## Abstract

**Background:**

Thyroid Cancer (TC) is the most common malignant disease of endocrine system, and its incidence rate is increasing year by year. Early diagnosis, management of malignant nodules and scientific treatment are crucial for TC prognosis. The first aim is the construction of a classification model for TC based on risk factors. The second aim is the construction of a prediction model for metastasis based on risk factors.

**Methods:**

We retrospectively collected approximately 70 preoperative demographic and laboratory test indices from 1735 TC patients. Machine learning pipelines including linear regression model ridge, Logistic Regression (LR) and eXtreme Gradient Boosting (XGBoost) were used to select the best model for predicting deterioration and metastasis of TC. A comprehensive comparative analysis with the prediction model using only thyroid imaging reporting and data system (TI-RADS).

**Results:**

The XGBoost model achieved the best performance in the final thyroid nodule diagnosis (AUC: 0.84) and metastasis (AUC: 0.72-0.77) predictions. Its AUCs for predicting Grade 4 TC deterioration and metastasis reached 0.84 and 0.97, respectively, while none of the AUCs for Only TI-RADS reached 0.70. Based on multivariate analysis and feature selection, age, obesity, prothrombin time, fibrinogen, and HBeAb were common significant risk factors for tumor progression and metastasis. Monocyte, D-dimer, T3, FT3, and albumin were common protective factors. Tumor size (11.14 ± 7.14 mm) is the most important indicator of metastasis formation. In addition, GGT, glucose, platelet volume distribution width, and neutrophil percentage also contributed to the development of metastases. The abnormal levels of blood lipid and uric acid were closely related to the deterioration of tumor. The dual role of mean erythrocytic hemoglobin concentration in TC needs to be verified in a larger patient cohort. We have established a free online tool (http://www.cancer-thyroid.com/) that is available to all clinicians for the prognosis of patients at high risk of TC.

**Conclusion:**

It is feasible to use XGBoost algorithm, combined with preoperative laboratory test indexes and demographic characteristics to predict tumor progression and metastasis in patients with TC, and its performance is better than that of Only using TI-RADS. The web tools we developed can help physicians with less clinical experience to choose the appropriate clinical decision or secondary confirmation of diagnosis results.

## Introduction

Thyroid Cancer (TC) is one of the most common malignant tumors of the endocrine system, with the ninth highest incidence worldwide, and three times the incidence in women than in men (1, 2). Neck ultrasound is the first choice for the clinical diagnosis and identification of TC, which can reveal smaller masses that are difficult to detect on palpation. Due to the complexity and overlapping nature of thyroid nodule echograms, ultrasound features may not be sufficient to accurately and reliably distinguish malignant tumors from benign nodules (3). Surgical resection is the primary treatment, and the extent of thyroidectomy depends on the pathologic type of TC and metastasis of lymph nodes (4). Although the majority of patients TC have a good prognosis, a subset of patients develops lymph node metastasis, whose five-year survival rate is substantially reduced. Regional lymph node metastasis in patients with differentiated thyroid cancer, especially of a papillary type, has been frequent. For example, papillary thyroid carcinoma (PTC) has a 24.1%-64.1% probability of developing central lymph node metastasis (CLNM) despite its slow progression, while the detection rate of CLNM by ultrasound is low (5, 6). The incidence of lateral cervical lymph node metastasis (LLNM) was second only to CLNM. American college of radiology thyroid imaging reporting and data system (ACR TI-RADS) has shown well concordance with in-needle aspiration and is recommended for clinical diagnosis of benign and malignant thyroid nodules (7). In the TI-RADS classification standard, there is only TI-RADS 4. However, the further detailed definitions are used in clinical practice, which is 4a (one suspicious US feature), 4b (two suspicious US features), 4c (three or four suspicious US features).

Obesity, smoking, and hormones are all possible causes of TC, and environmental factors, especially ionizing radiation, are currently considered to be recognized risk factors for TC (8). Early diagnosis, management of malignant nodules and scientific treatment are crucial for TC prognosis. More and more attention has been paid to the investigation of the influencing factors of prognosis (9). As medical database management improves, medical information is integrated and standardized, and computing power increases, artificial intelligence (AI), especially ML and deep learning, grows in its potential use in medicine (10–14) Also, in many countries, including China, Clinical follow-up observation is often used for thyroid nodules with TI-RADS 3, and surgery is often used for TI-RADS 5 and above. The choice of treatment for TI-RADS 4 (especially TI-RADS 4a) is controversial. Therefore, we focused on the prediction of thyroid nodules with TI-RADS 4. Timely and effective evaluation of preoperative risk factors allows accurate differentiation of benign and malignant thyroid nodules and screening of patients most likely to metastasize. In general, the first aim is the construction of a classification model for TC based on risk factors. Our second aim is the construction of a prediction model for metastasis based on risk factors.

## Materials and methods

### Participants

This retrospective study was approved by the Ethics Committee of Shanghai Ruijin Rehabilitation Hospital. All the procedures were implemented based on the principles of the Declaration of Helsinki. Since this is a retrospective research and anonymized data were evaluated, patient consent was waived by our institutional ethic committee. Each patient underwent ultrasonography before surgery by the same group of five ultrasound specialists (more than five years of clinical experience). The TI-RADS classification was performed according to the Guidelines for the diagnosis and treatment of thyroid cancer (National Health Commission of the People’s Republic of China, 2018.). All patients basically undergo US-FNAB before the surgery, except for the following cases, huge thyroid nodule (the diameter > 3 cm), sign of tracheal compression and typical sign of lymph node metastasis. All patients were diagnosed by pathologists based on preoperative and postoperative specimens. Malignant patients with non-papillary carcinomas have been excluded based on postoperative pathological findings. Most patients’ diagnosis and treatment process is shown in **Supplementary Figure 1**. The target being predicted in this paper included, benign/malignant thyroid nodules, benign/malignant thyroid nodules defined as TI-RADS 4 or as TI-RADS 4a, central or lateral lymph node metastases in the neck, etc. In addition, in our study, the prediction targets are represented in binary (0: negative, 1: positive). In the model construction of this study, we used the Synthetic Minority Oversampling Technique (SMOTE) algorithm to balance the training set. Meanwhile, AUC calculation of TI-RAD score/level is based on the order grade of TI-RAD ranking, and detailed stratification is shown in **Supplementary Table 1**.

### Data pre-processing and feature selection

Our structured database initially contains approximately 70 clinical variables (**Supplementary Table 2**). First, features with more than 30% missing were excluded. Similarly, patients with missing features higher than this threshold would also be removed from model construction Then, the missing values of the categorical variables were filled by the mode, and the continuous variables were filled by the Missforest method (15). In order to reduce the influence of the range difference of the features on the model construction, the noncategorical data was processed by mean and SD. Categorical data were further transformed into binary dummy variables.

We used the LassoCV algorithm, which employs the leave-one-out method, and then calculates the optimal λ according to the algorithm formula (16). The purpose of feature selection is to determine the best subset of features that can be used to predict each outcome variable. We used machine learning method lasso regularization to construct feature subsets. The predicted outcomes were benign or malignant diagnosis and metastasis. The result diagram of each category includes AUC curve, PR curve, and factor importance diagram after feature screening of Lasso. The top features selected by lasso which contributed to each outcome was analyzed by using unpaired Student’s t-test or Mann-Whitney U test for continuous variables and Chi-squared or Fisher’s exact test for categorical variables between the positive and negative group, as appropriate.

### Development of ML-based models

**Figure 1:**
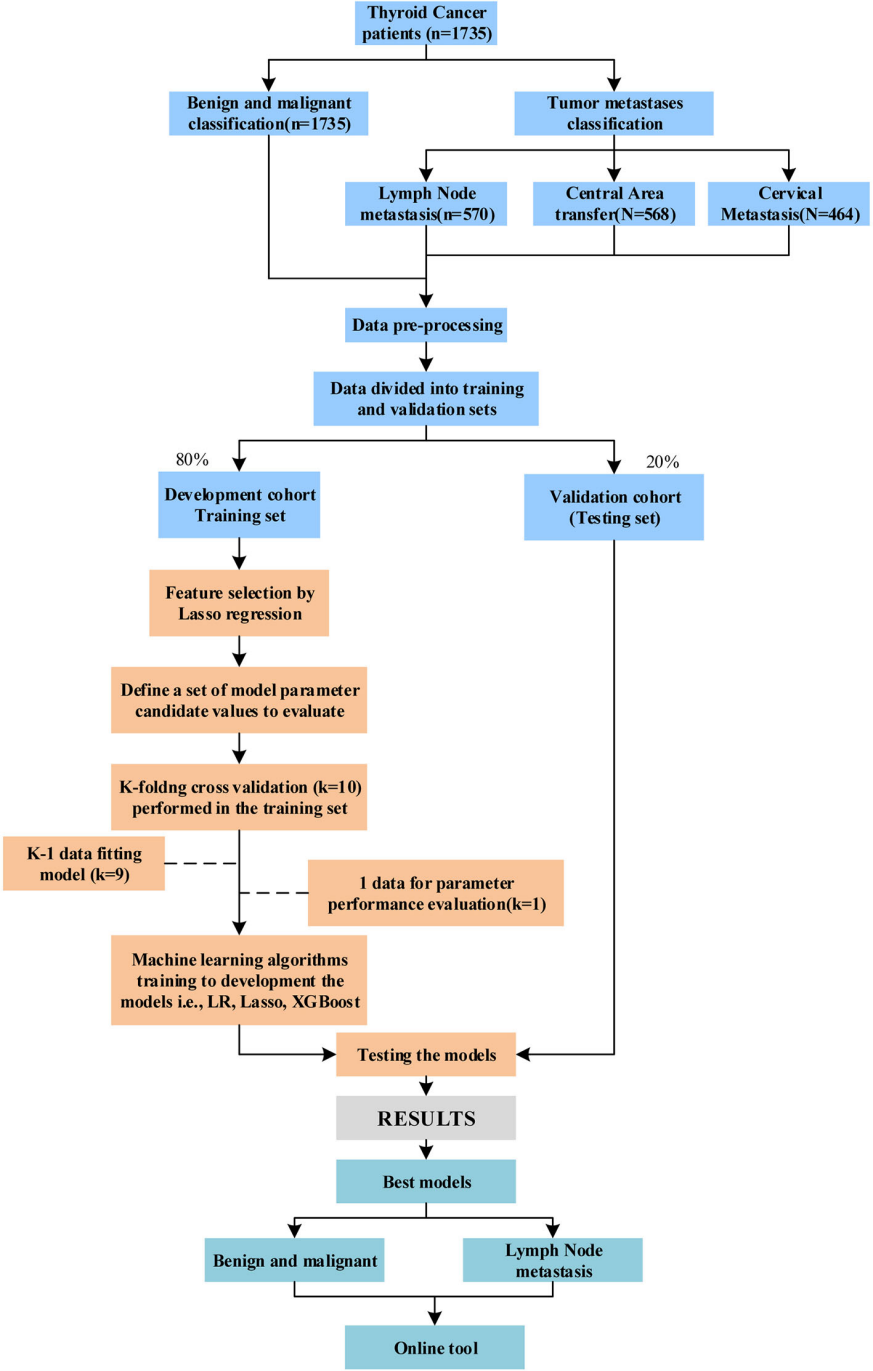
Study flowchart.

Model development includes linear model Ridge, LR and XGBoost machine learning models (17–19). The model was trained in the training set using 10-fold cross-validation and the grid search method was used to adjust the parameters of each algorithm. Hyper-parameter tuning range of each machine learning algorithm is shown in **Supplementary Table 3**. In this procedure, the training data set is randomly divided into 10 equal folds, each containing the same number of events. Ten validation experiments are then performed, with each fold used in rotation as the validation set, and the remaining 9 folds as the training set. Therefore, each hyperparameter combination was trained and tested for 10 times, the average values of 10 experimental models were calculated to select the optimal global hyperparameter measurement results. In order to quantify the model’s discrimination, a test set was applied to evaluate the model. Finally, we developed an online tool for XGBoost-based malignant tumor TC diagnosis and metastasis. We used TI-RADS alone to predict it for comprehensive comparison. The data set was divided according to TI-RADS, including two categories, namely, TI-RADS patients with 4a and all category 4 of TI-RADS. **Figure 1** showed the study flowchart.

### Statistical analysis

Continuous variables are expressed as mean ± SD for normally distributed data. Categorical variables are presented as frequency. Continuous variables were compared using analysis of two-tailed Student’s t-tests for normally distributed data, and the Mann–Whitney U-test or Kruskal–Wallis H-test for non-normally distributed data. Categorical variables were compared using the Chi-square test or the Fisher exact test. Test for the assumptions of normality distribution and variance homogeneity have been performed properly. Statistical analysis was performed with R platform. *P*-values< 0.05 were considered statistically significant. The evaluation index of categorical dependent variable includes AUC, sensitivity (recall), specificity, accuracy, FP Rate, precision, AP (Average precision), and F1. In addition, the factor weight of the linear model is taken as the Importance of the factor.

## Results

### Patients’ characteristics

As shown in **Table 1**, our retrospective study cohort consisted of 1304 (75.16%, including 691 TC and 613 benign thyroid nodules) females and 431 (24.84%, including 247 malignant TC and 184 benign thyroid nodules) males, with a mean age of 44.58 ± 13.09 years, mean BMI of 23.33 ± 3.45.

**Table 1:** Baseline characteristics of included patients.

	Total (n=1735)	Malignant (n=938)	Benign (n=797)	P-value
**Age**				
Mean (SD)	44.58 ± 13.09	43.29 ± 12.72	46.10 ± 13.35	<0.01
**BMI**				
Number of subjects	1668	895	773	
Mean (SD)	23.33 ± 3.45	23.54 ± 3.70	23.08 ± 3.12	<0.01
Missing	67 (3.9%)	43 (4.6%)	24 (3.0%)	
**Gender**				
Number of subjects	1734	938	796	
Female	1304 (75.16 %)	691 (73.67 %)	613 (76.91 %)	0.12
Male	431 (24.84 %)	247 (26.33 %)	184 (23.09 %)	
**Height**				
Number of subjects	1671	896	775	
Mean (SD)	163.61 ± 7.62	163.78 ± 8.01	163.41 ± 7.14	0.31
Missing	64 (3.7%)	42 (4.5%)	22 (2.8%)	
**Weight**				
Number of subjects	1678	902	776	
Mean (SD)	62.64 ± 11.39	63.31 ± 11.78	61.86 ± 10.88	<0.01
Missing	57 (3.3%)	36 (3.8%)	21 (2.6%)	
**TSH**				
Number of subjects	1479	792	687	
Mean (SD)	2.32 ± 3.06	2.35 ± 1.95	2.27 ± 3.98	0.64
Missing	256 (14.8%)	146 (15.6%)	110 (13.8%)	
**FT3**				
Number of subjects	1470	787	683	
Mean (SD)	4.94 ± 0.75	4.89 ± 0.72	5.00 ± 0.77	<0.01
Missing	265 (15.3%)	151 (16.1%)	114 (14.3%)	
**FT4**				
Number of subjects	1469	786	683	
Mean (SD)	17.52 ± 3.65	17.49 ± 2.71	17.55 ± 4.49	0.78
Missing	266 (15.3%)	152 (16.2%)	114 (14.3%)	
**PTH**				
Number of subjects	1465	784	681	
Mean (SD)	5.95 ± 9.85	5.57 ± 3.72	6.39 ± 13.88	0.14
Missing	270 (15.6%)	154 (16.4%)	116 (14.6%)	
**Ca**				
Number of subjects	1575	847	728	
Mean (SD)	2.43 ± 0.26	2.43 ± 0.33	2.43 ± 0.14	0.58
Missing	160 (9.2%)	91 (9.7%)	69 (8.7%)	

### Predictive performance of ML-based models

Predictive models for TC determining benign/malignant and types of metastasis were developed based on these algorithms. The predictive performance of the models is shown in **Table 2**, including AUC, sensitivity, specificity, accuracy, FP Rate, precision, AP, and F1. There are significant performance differences between the different models. All models we developed performed significantly better than only TI-RADS. The AUC value of XGBoost models is the most prominent in terms of all prediction (all AUC values above 0.70).

**Table 2:** Performance summary.

Models	AUC	95%CI		sensitivity (recall)	specificity	accuracy	FP rate	precision	AP	F1
		Lower bound	Upper bound							
**Benign or Malignant**										
LR	0.82	0.77	0.86	0.86	0.69	0.78	0.31	0.76	0.83	0.81
Ridge	0.82	0.77	0.86	0.83	0.72	0.78	0.28	0.78	0.80	0.80
XGBoost	0.84	0.80	0.88	0.76	0.79	0.77	0.21	0.81	0.85	0.78
**Lymph Node Metastasis**										
LR	0.68	0.58	0.78	0.43	0.88	0.70	0.12	0.71	0.64	0.54
Ridge	0.69	0.58	0.79	0.67	0.72	0.70	0.28	0.62	0.65	0.65
XGBoost	0.72	0.62	0.82	0.72	0.69	0.70	0.31	0.61	0.66	0.66
**Central Area Metastasis**										
LR	0.67	0.57	0.77	0.84	0.49	0.63	0.51	0.52	0.55	0.64
Ridge	0.67	0.57	0.77	0.91	0.41	0.61	0.59	0.50	0.56	0.65
XGBoost	0.72	0.62	0.82	0.67	0.72	0.70	0.28	0.61	0.66	0.64
**Cervical Metastasis**										
LR	0.76	0.57	0.95	0.80	0.77	0.77	0.23	0.30	0.35	0.43
Ridge	0.75	0.56	0.94	0.80	0.67	0.69	0.33	0.23	0.37	0.36
XGBoost	0.77	0.57	0.96	0.80	0.69	0.70	0.31	0.24	0.55	0.36
**Benign / Malignant_All 4 (4a + 4b + 4c)**										
LR	0.78	0.75	0.82	0.67	0.76	0.71	0.24	0.8	0.84	0.73
Ridge	0.78	0.75	0.82	0.66	0.77	0.71	0.23	0.81	0.83	0.73
XGBoost	0.84	0.81	0.87	0.75	0.79	0.77	0.21	0.84	0.88	0.79
**Benign / Malignant_4a**										
LR	0.72	0.67	0.77	0.74	0.63	0.68	0.37	0.58	0.61	0.65
Ridge	0.72	0.67	0.77	0.72	0.62	0.66	0.38	0.56	0.61	0.63
XGBoost	0.78	0.73	0.82	0.86	0.57	0.68	0.43	0.57	0.67	0.69
**Metastasis_All 4 (4a + 4b + 4c)**										
LR	0.82	0.77	0.88	0.81	0.74	0.77	0.26	0.72	0.8	0.76
Ridge	0.81	0.76	0.87	0.78	0.72	0.75	0.28	0.70	0.79	0.74
XGBoost	0.84	0.78	0.89	0.74	0.80	0.78	0.20	0.76	0.82	0.75
**Metastasis_4a**										
LR	0.95	0.91	0.99	1.00	0.8.	0.90	0.20	0.83	0.94	0.91
Ridge	0.94	0.89	0.99	0.96	0.84	0.90	0.16	0.86	0.92	0.91
XGBoost	0.97	0.95	1.00	0.96	0.92	0.94	0.08	0.92	0.97	0.94
**Only TI-RADS**										
Benign or Malignant	0.79	0.75	0.84	0.76	0.73	0.75	0.27	0.77	0.76	0.77
Lymph Node Metastasis	0.50	0.41	0.60	0.89	0.15	0.45	0.85	0.41	0.40	0.56
Central Area Metastasis	0.55	0.45	0.64	0.93	0.22	0.50	0.78	0.44	0.41	0.59
Cervical Metastasis	0.62	0.49	0.75	0.89	0.41	0.46	0.59	0.14	0.12	0.24
Benign / Malignant_All4	0.69	0.66	0.72	0.66	0.72	0.68	0.28	0.77	0.71	0.71
Benign / Malignant_4a	0.50	0.49	0.51	1.00	0.00	0.41	1.00	0.41	0.40	0.58
Metastasis_All4	0.64	0.57	0.70	0.3	0.89	0.62	0.11	0.70	0.56	0.42
Metastasis_4a	0.50	0.47	0.53	1.00	0.00	1.00	0.50	0.50	0.67	1.00

#### Predictive model for distinguish between benign and malignant

**Figure 2:**
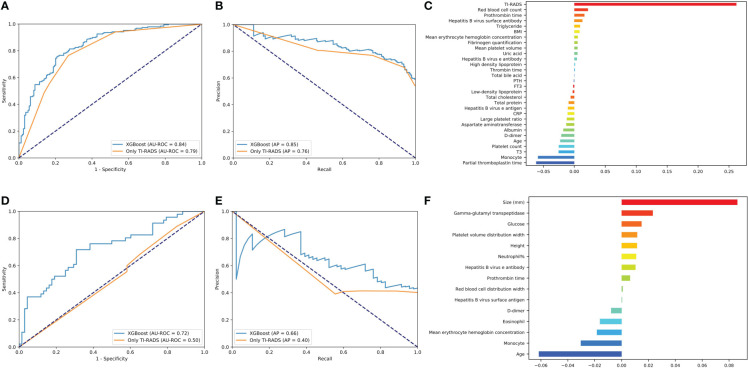
Evaluation of the malignant and lymph node metastasis predictive models. **(A, D)** The average ROC curves from of XGBoost and Only TI-RADS models in the validation sets. **(B, E)** The average PR curves, indicating the tradeoff between precision and recall. **(C, F)** The histogram describes the important features of the XGBoost predictive model for malignant and lymph node metastasis.

All models have excellent performance in benign/malignant prediction, and the accuracy rate is up to 0.75. Among them, XGBoost obtains the highest AUC value of 0.84, and the accuracy rate is 0.77 (**Figure 2A**). We use the AP value as the criterion for the PR curve. AP value, a weighted mean of precisions when achieved at a certain threshold.


$$AP=\sum_{n}(R_{n}-R_{n-1})P_{n}$$

Where Pn (precision) and Rn (recall) are at the nth threshold. (Rk, Pk) denotes an operating point.

It can be seen that the aPs of XGBoost models is 0.85 in benign/malignant prediction (**Figure 2B**). The confusion matrix (rounding) was also calculated for these models (**Table 3**). After a comprehensive comparison, we think XGBoost is more suitable for TC prediction. Feature importance was calculated by the sum of the decrease in error when split by a variable, reflecting each variable’s contribution to prediction. The important features of the predictive model for benign/malignant, as were shown in **Figure 2C** and **Supplementary Figure 2**. The features, such as high-density lipoprotein, fibrinogen quantification, BMI, and hepatitis B virus surface antibody are risk factors (*P*< 0.05). The protective factors include T3, age, monocyte, hepatitis B virus e antibody, aspartate aminotransferase, FT3, and D dimer (*P*< 0.05).

**Table 3:** Confusion matrices of predictive models.

Model	Actual	Predictive	
		Negative	Positive
**Benign or Malignant**			
LR	Negative	109	50
	Positive	26	162
Ridge	Negative	114	45
	Positive	32	156
XGBoost	Negative	126	33
	Positive	46	142
**Lymph Node Metastasis**			
LR	Negative	60	8
	Positive	26	20
Ridge	Negative	49	19
	Positive	15	31
XGBoost	Negative	47	21
	Positive	13	33
**Central Area Metastasis**			
LR	Negative	34	35
	Positive	7	38
Ridge	Negative	28	41
	Positive	4	41
XGBoost	Negative	50	19
	Positive	15	30
**Cervical Metastasis**			
LR	Negative	64	19
	Positive	2	8
Ridge	Negative	56	27
	Positive	2	8
XGBoost	Negative	57	26
	Positive	2	8
**Benign/Malignant_All 4 (4a + 4b + 4c)**			
LR	Negative	246	79
	Positive	152	315
Ridge	Negative	251	74
	Positive	159	308
XGBoost	Negative	258	67
	Positive	118	349
**Benign/Malignant_4a**			
LR	Negative	147	86
	Positive	41	118
Ridge	Negative	145	88
	Positive	45	114
XGBoost	Negative	132	101
	Positive	23	136
**Metastasis_All 4 (4a + 4b + 4c)**			
LR	Negative	94	33
	Positive	20	85
Ridge	Negative	92	35
	Positive	23	82
XGBoost	Negative	102	25
	Positive	27	78
**Metastasis_4a**			
LR	Negative	40	10
	Positive	0	50
Ridge	Negative	42	8
	Positive	2	48
XGBoost	Negative	46	4
	Positive	2	48
**Only TI-RADS**			
Benign or Malignant	Negative	116	43
	Positive	44	143
Lymph Node Metastasis	Negative	10	57
	Positive	5	40
Central Area Metastasis	Negative	15	53
	Positive	3	41
Cervical Metastasis	Negative	34	49
	Positive	1	8
Benign/Malignant_All 4	Negative	233	92
	Positive	159	308
Benign/Malignant_4a	Negative	0	233
	Positive	0	159
Metastasis_All 4	Negative	113	14
	Positive	73	32
Metastasis_4a	Negative	0	50
	Positive	0	50

#### Predictive model for lymph node metastasis

XGBoost obtains the highest AUC value of 0.72, and the AP is 0.66 in lymph node metastasis prediction (**Figures 2D, E** **)**. As shown in **Figure 2F** and **Supplementary Figure 3**, size, neutrophil, hepatitis B virus e antibody, and prothrombin time all increase the likelihood of lymph node metastasis (*P*< 0.05). On the contrary, age, monocyte, and eosinophil reduces this possibility (*P*< 0.05).

#### Predictive model for central area metastasis

**Figure 3:**
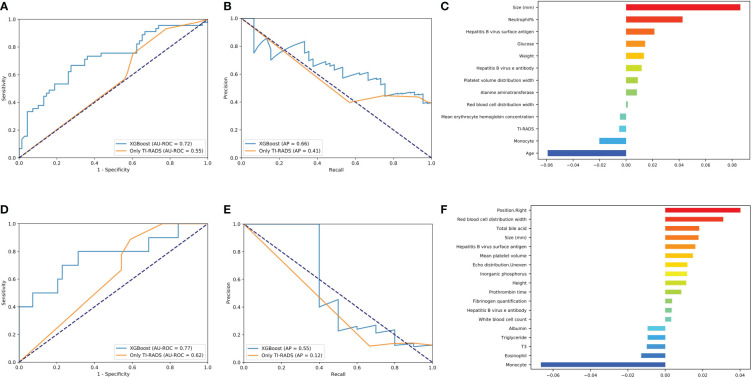
Evaluation of the central area and cervical metastasis predictive models. **(A, D)** The average ROC curves from of XGBoost and Only TI-RADS models in the validation sets. **(B, E)** The average PR curves, indicating the tradeoff between precision and recall. **(C, F)** The histogram describes the important features of the XGBoost predictive model for central area and cervical metastasis.

XGBoost obtains the highest AUC value of 0.72, and the AP is 0.66 in lymph node metastasis prediction (**Figures 3A, B** **)**. As shown in **Figure 3C** and **Supplementary Figure 4**, size, neutrophil, weight, and hepatitis B virus e antibody all increase the likelihood of lymph node metastasis (*P*< 0.05). On the contrary, age and monocyte reduces this possibility (*P*< 0.05).

#### Predictive model for cervical metastasis

XGBoost obtains the highest AUC value of 0.77, and the AP is 0.55 in lymph node metastasis prediction (**Figures 3D, E** **)**. TC patients with cervical metastasis tend to have an uneven echo distribution. and is more likely to develop right cervical lymphatic metastases (**Supplementary Figure 5A**). As shown in **Figure 3F** and **Supplementary Figures 5B, C**, total bile acid, size, and prothrombin time all increase the likelihood of lymph node metastasis (*P*< 0.05). On the contrary, monocyte, eosinophil, T3, and albumin reduces this possibility (*P*< 0.05).

#### Predictive model for benign/malignant and metastasis in classification All 4 (4a + 4b + 4c) and 4a

**Figure 4:**
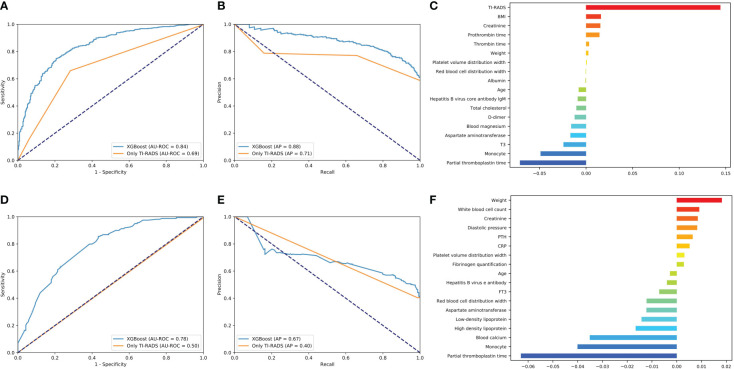
Predictive models of evaluation of the benign/malignant nodules of all 4^th^ category of TI-RADS and only 4th category. **(A, D)** The average ROC curves from of XGBoost and Only TI-RADS models in the validation sets. **(B, E)** The average PR curves, indicating the tradeoff between precision and recall. **(C, F)** The histogram describes the important features of the XGBoost predictive model for benign/malignant nodules of all 4^th^ category of TI-RADS.

**Figure 5:**
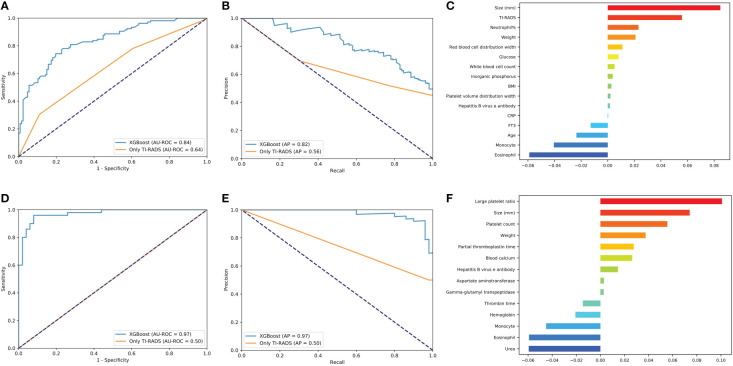
Predictive models of metastasis evaluation for benign/malignant nodules of all 4^th^ category of TI-RADS and only 4th category**. (A, D)** The average ROC curves from of XGBoost and Only TI-RADS models in the validation sets. **(B, E)** The average PR curves, indicating the tradeoff between precision and recall. **(C, F)** The histogram describes the important features of the XGBoost predictive model for metastasis in all 4^th^ category of TI-RADS and only 4th category.

The predictive power of benign/malignant and metastasis for TI-RADS 4a and all category 4 of TI-RADS was developed based on the three algorithms (all AUC values above 0.70) and was better than that identified by TI-RADS score alone (all AUC values below 0.70) (**Figures 4**, **5**). The performance of XGBoost is still in the best position, and the AUC in predicting metastasis 4a even reaches 0.97. In the model for predicting benign/malignant in all category 4 of TI-RADS, the TI-RADS category was helpful in identifying tumor progression, followed by BMI, creatinine, prothrombin time, and thrombin time. When the ML model is trained, the AUC is affected by the inclusion of TIRADS in the features used. The AUC is improved when the predictors include TIRADS. Partial thromboplastin time, monocyte, and T3 were negatively correlated factors (**Figure 4C**). In the model for predicting benign/malignant in 4a category, the positive correlation factors were white blood cell count, creatinine, diastolic pressure, and the negative correlation factor were partial thromboplastin time, monocyte, blood calcium, lipoprotein etc. (**Figure 4F**). In the model for predicting metastasis in all category 4 of TI-RADS, the tumor size, TI-RADS, and neutrophil% were risk factors in tumor metastasis. Eosinophil, monocyte, age, and FT3 were negatively correlated factors (**Figure 5C**). In the model for predicting metastasis in 4a category, the large platelet ratio, size, and platelet count were risk factors in tumor metastasis. Urea, eosinophil, monocyte, hemoglobin, and thrombin time were negatively correlated factors (**Figure 5F**).

### Online tool for XGBoost-based malignant tumor diagnosis and metastasis

**Figure 6:**
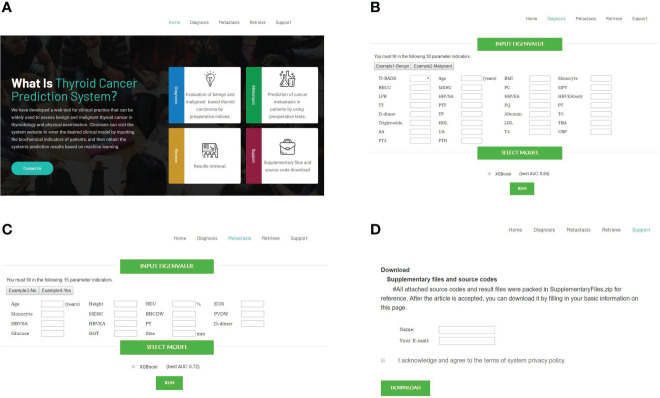
Web-based tools for diagnosis of malignant and metastasis TC using preoperative laboratory tests and demographic characteristics (http://www.cancer-thyroid.com/). **(A)** Main page. **(B)** Diagnosis page. **(C)** Metastasis page. **(D)** Supplementary files and source codes.

We developed an online tool (Cancer-thyroid.com) for diagnosis of malignant thyroid cancer using biochemical indicators (**Figure 6**). It is available for free at http://www.cancer-thyroid.com/. By input patients’ preoperative eigenvalue can diagnose TC malignancy (AUC: 0.84) and metastasis (AUC: 0.72) with excellent accuracy.

## Discussion

In this study, we collected about 70 kinds of pre-operational indicators from 1735 patients with thyroid nodules, including demographics, pre-operational blood tests, and ultra sound data. Three ML models were developed and compared, namely Ridge, LR, and XGBoost. The XGBoost model achieved the best performance in the final TC benign/malignant diagnosis (AUC: 0.84) and metastasis (AUC: 0.72-0.77) predictions. We also compared ML prediction with Only TI-RADS for the identification of high-risk thyroid nodules. We believe that the XGBoost-based model is more convincing in predicting people at high risk for TC because its AUCs for predicting Grade 4 TC progression and metastasis reached 0.84 and 0.97, respectively, while none of the AUCs for Only TI-RADS reached 0.70. XGBoost as an open-source package, has shown excellent and consistent performance in several recent ML prediction disease and data mining challenges, such as COVID-19 mortality risk (20), oropharyngeal cancer recurrence risk prediction (21), etc. The observed model performance suggested that it was feasible to derive effective ML prediction models of TC progression diagnosis, which may play a significant role in predicting etiologies of patients with suspected malignant tumors. In addition, we have developed a web tool (http://www.cancer-thyroid.com/) for clinical practice that can be widely used to assess benign and malignant thyroid nodules (Only For Research Use). Clinicians can visit the system website to enter the desired clinical model by inputting the biochemical indicators of patients, and then obtain the system’s prediction results based on ML.

Statistical analysis of the electronic medical records included in this study revealed that the risk of TC was approximately three times higher in women than in men, which is consistent with previous reports (22). Although the deterioration and metastasis of TC did not seem to have gender difference, it was associated with obesity (BMI: 23.54 ± 3.70), and tended to be younger (43.29 ± 12.72). Prothrombin time (PT), fibrinogen quantification, hepatitis B virus e antibody (HBeAb) were also positively correlated with tumor progression and metastasis. Monocyte, D-dimer, T3, FT3 and albumin were the common protective factors to prevent the deterioration and metastasis of TC. The link between thyroid disorders and hemostatic system is well known and well established (23). Fibrinogen is an important protein involved in coagulation and hemostasis. The presence of fibrinogen can affect the growth and metastasis of malignant tumor cells. It has been known that the assessment of plasma fibrinogen and fibrinolytic products is helpful for cancer diagnosis, treatment effect and prognosis (24). Studies have shown that cancer patients with HBeAb^+^ show unique clinical characteristics, which is an independent risk factor affecting the prognosis of the disease (25). Levothyroxine suppression treatment for benign thyroid nodules alters coagulation showed that D-dimer level increased after treatment (26).

Tumor size (11.14 ± 7.14mm) is the most significant index of metastasis. In addition, gamma glutamyl transpeptidase (GGT), glucose, platelet volume distribution width and neutral% are also important risk factors. GGT levels have been shown in several studies to play a role in cancer progression and to be an important poor prognostic factor, such as increasing the metastatic growth of B16 melanoma cells in the mouse liver and increasing the likelihood of recurrence after hepatectomy in patients with liver metastases from colorectal cancer (27, 28). Neutrophils create a fertile soil for metastasis, but how they are activated at the site of metastasis still needs to be explored (29).

Triglyceride and uric acid (UA) are unique risk indicators for malignant TC tumors. Aspartate aminotransferase is a unique protective indicator of TC deterioration. The level of blood lipid is related to the outcome of many kinds of cancer, and TC patients also have dyslipidemia (30). Binary logistic regression demonstrated that female differentiated thyroid cancer patients had higher risks of developing hyperlipidemia than male patients (31). UA is the end product of purine metabolism in the human body which has been found to be one of the most important parts of metabolic syndrome. It is closely related to coronary heart disease, type 2 diabetes mellitus, and hypertension (32). Interestingly, the increase of mean erythrocytic hemoglobin concentration indicates a high probability of TC deterioration, but it is a protective factor of metastasis. Whether mean erythrocytic hemoglobin concentration has a dual role in TC needs to be verified in a larger patient cohort.

This study also has some limitations: first, because the data are from the same database and the patients are all Chinese, there may be potential bias. Secondly, a small number of patients lost part of the information. Finally, the prognosis and survival of patients with TC were not discussed in this study, and our team will make further analysis in the future plan.

## Conclusion

The advantage of this study is that ML model is used to predict the possibility of TC deterioration and transfer for the first time. Several most important risk and protective factors are listed and compared with traditional TI-RADS. At the same time, a free online tool for all clinicians to predict patients with high TC risk has been established, which can act as a virtual assistant to improve the efficiency and accuracy of diagnosis.

## Data availability statement

The raw data supporting the conclusions of this article will be made available by the authors, without undue reservation. Please contact corresponding authors for more data information.

## Ethics statement

The studies involving human participants were reviewed and approved by The Ethics Committee of Shanghai Ruijin Rehabilitation Hospital. Written informed consent for participation was not required for this study in accordance with the national legislation and the institutional requirements.

## Author contributions

JG, RX, YZ, and SZ collected and analyzed data and wrote the manuscript. ZZ, DX, MD, TL, WX, ZN, EM, and DT assisted with the data presentation and manuscript writing. JF, DS, and SZ contributed in the study design, data analysis and manuscript writing. All authors contributed to the article and approved the submitted version.
