# Supplementary figures and images for: A machine learning-based approach to predicting the malignant and metastasis of thyroid cancer
Source: Front Oncol. 2022 Dec 19;12:938292. doi: 10.3389/fonc.2022.938292 (PMC9806162; doi:10.3389/fonc.2022.938292)

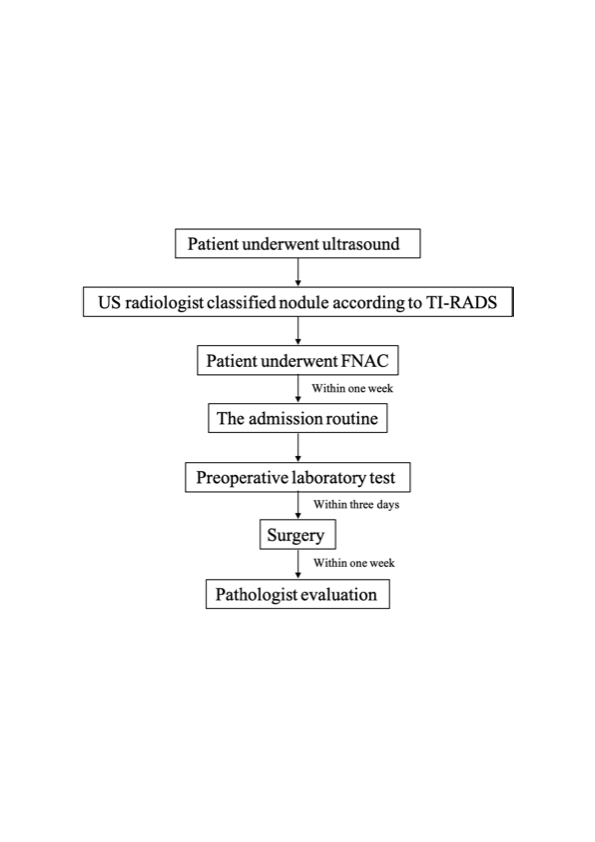

Supplement: Supplementary file 1 [file Image_1.tiff]

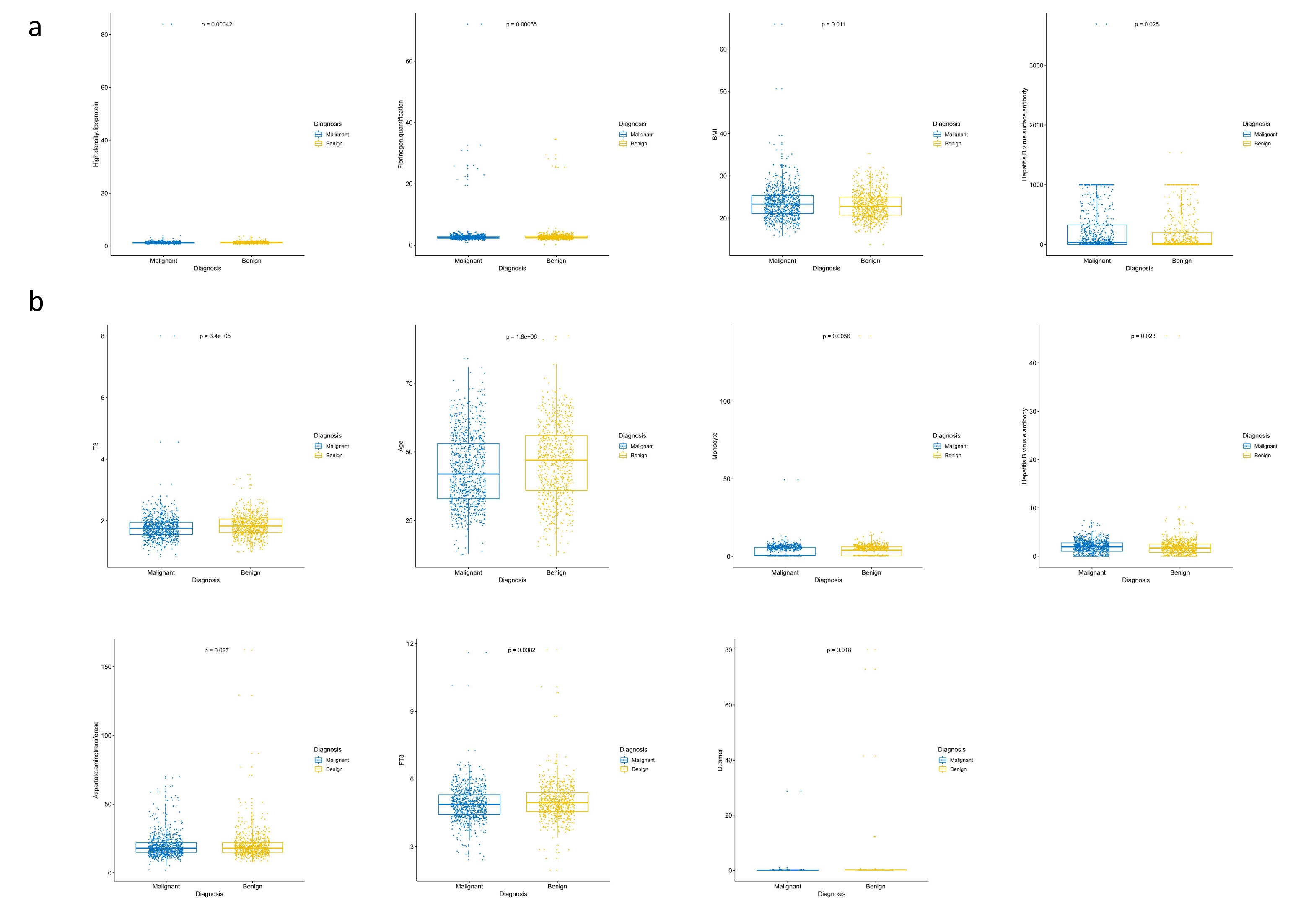

Supplement: Supplementary file 2 [file Image_2.jpg]

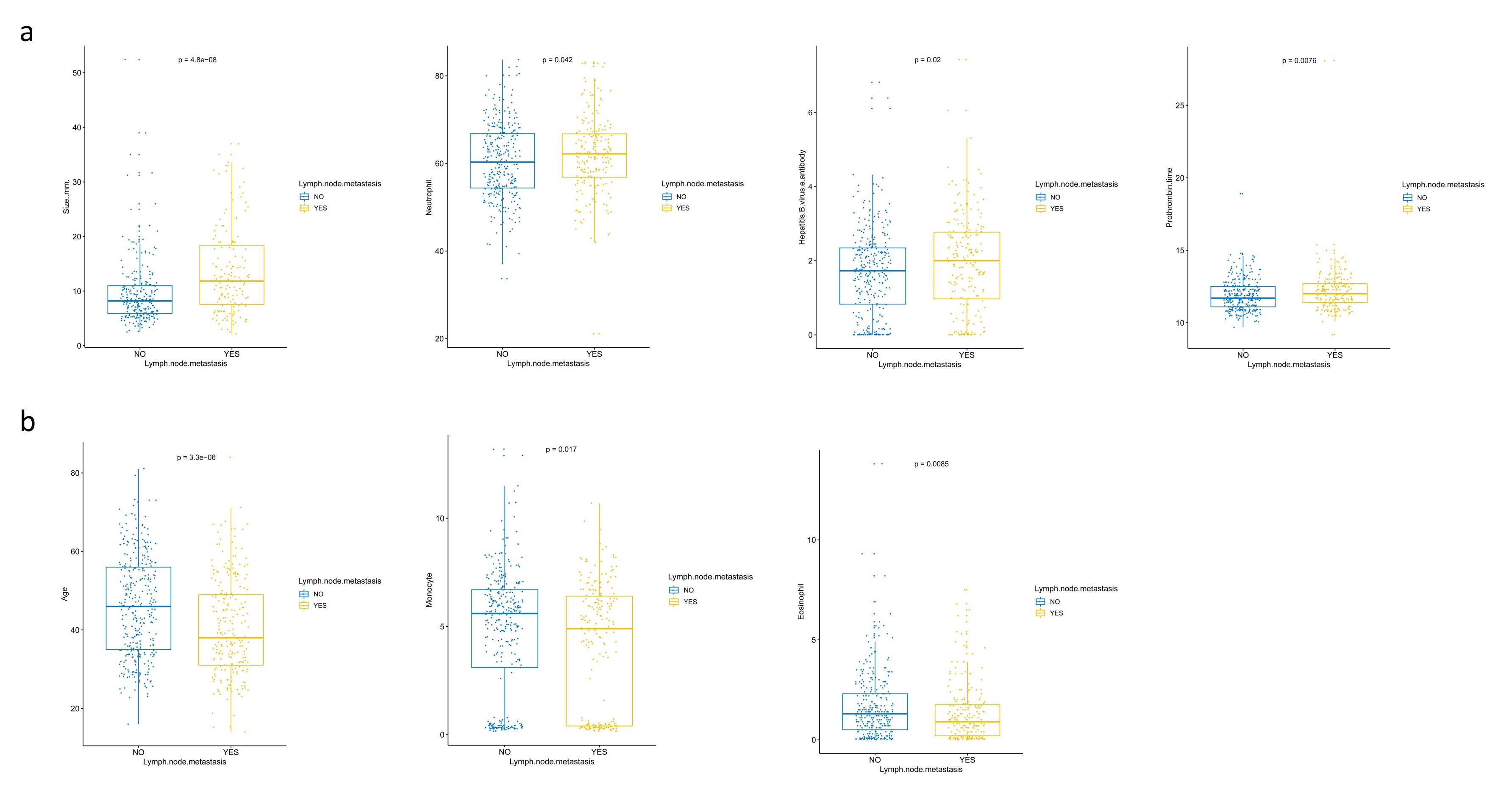

Supplement: Supplementary file 3 [file Image_3.jpg]

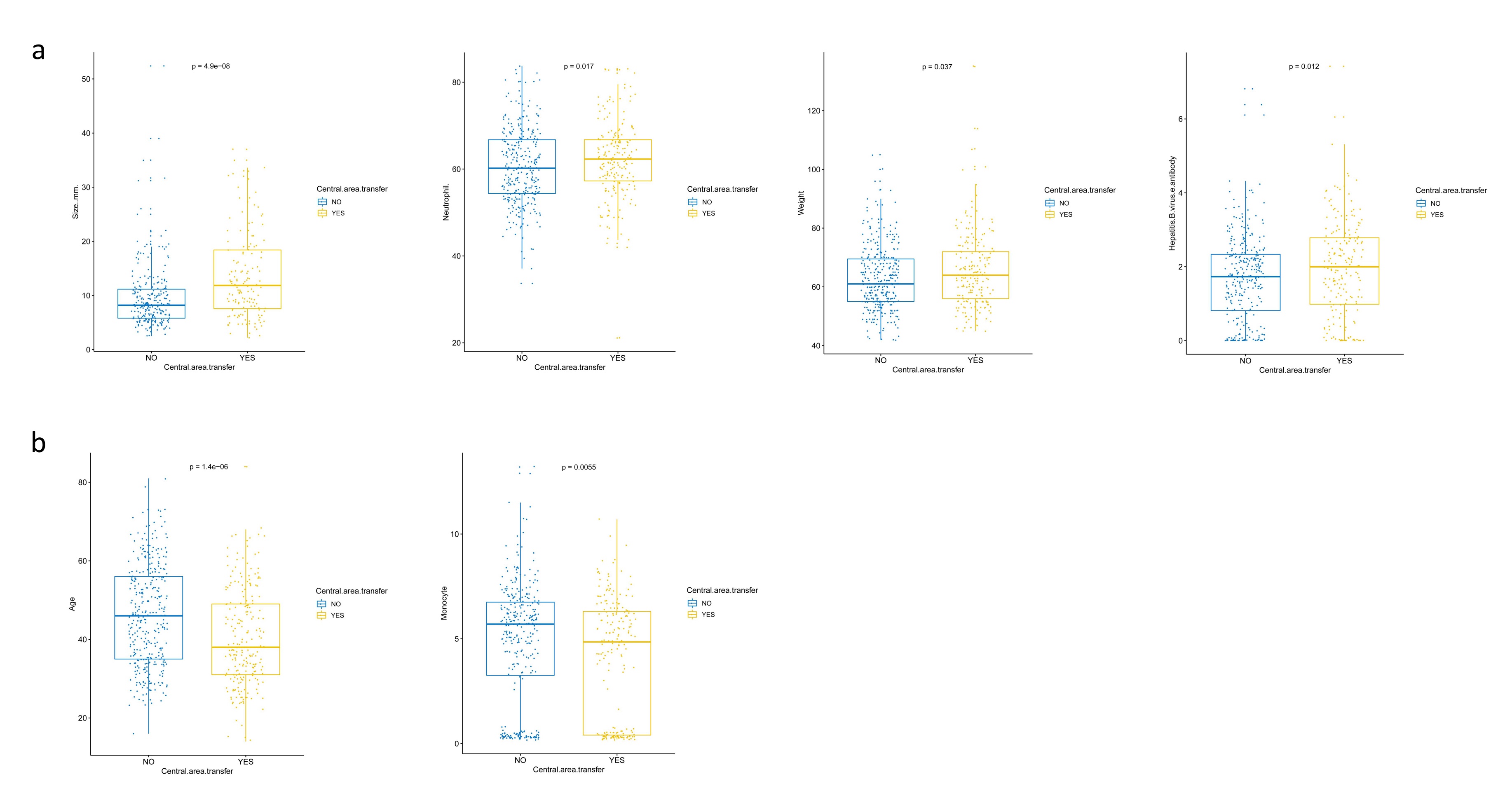

Supplement: Supplementary file 4 [file Image_4.jpg]

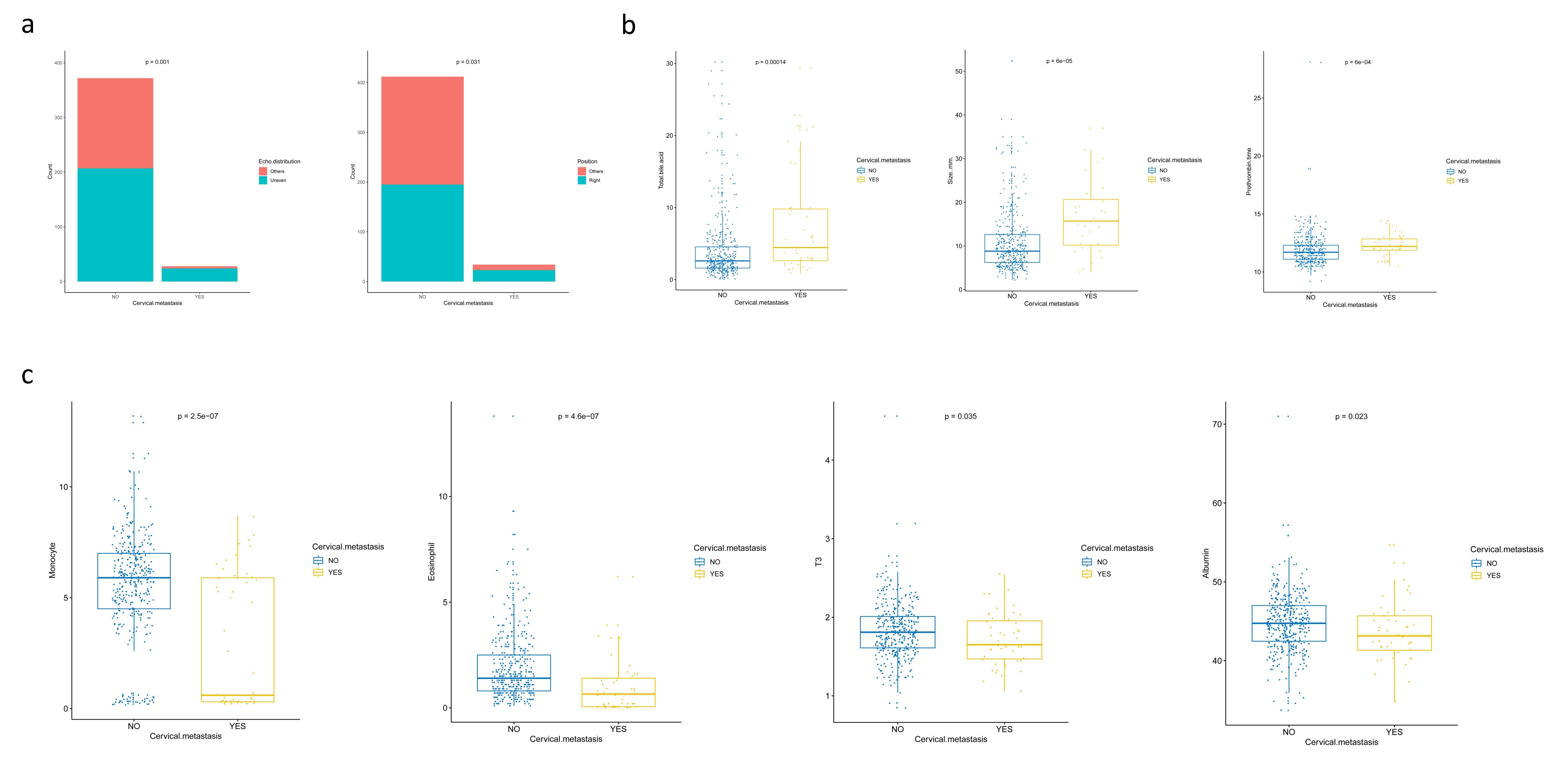

Supplement: Supplementary file 5 [file Image_5.jpg]
