# Supplementary material for: A machine learning-based approach to predicting the malignant and metastasis of thyroid cancer
Source: Front Oncol. 2022 Dec 19;12:938292. doi: 10.3389/fonc.2022.938292 (PMC9806162; doi:10.3389/fonc.2022.938292)
Supplement: Supplementary file 6 [file Table_1.docx]

Supplementary table 1. The Ranking methods of TI-RAD score/level

| TI-RADS | Ranking |
| --- | --- |
| GROUP2 | 1 |
| GROUP2-3 | 2 |
| GROUP3 | 3 |
| GROUP3-4A | 4 |
| GROUP4 | 5 |
| GROUP4A | 6 |
| GROUP4B | 7 |
| GROUP4C | 8 |
| GROUP5 | 9 |
| GROUP5A | 10 |
| GROUP5C | 11 |
| GROUP6 | 12 |
