# Supplementary material for: A machine learning-based approach to predicting the malignant and metastasis of thyroid cancer
Source: Front Oncol. 2022 Dec 19;12:938292. doi: 10.3389/fonc.2022.938292 (PMC9806162; doi:10.3389/fonc.2022.938292)
Supplement: Supplementary file 7 [file Table_2.docx]

Supplementary table 2. The 70 clinical variables and features

Age, BMI, Gender, Systolic pressure, ,Diastolic pressure, Height, Weight, Self-exposed hypertension, Measuring Hypertension, Self-reported diabetes, White blood cell count, Neutrophil%, Lymphocyte%, Eosinophil, Basophils, Monocyte, Red blood cell count, Hemoglobin, Mean erythrocyte hemoglobin concentration, Red blood cell distribution width, Platelet count, Mean platelet volume, Platelet volume distribution width, Large platelet ratio, Hepatitis B virus surface antigen, Hepatitis B virus surface antibody, Hepatitis B virus e antigen, Hepatitis B virus e antibody, Hepatitis B virus core antibody lgM, Thrombin time, Partial thromboplastin time, Fibrinogen quantification, Prothrombin time, D-dimer, Total protein, Albumin, Glucose, Total cholesterol, Triglyceride, High density lipoprotein, Low-density lipoprotein, Blood calcium, Inorganic phosphorus, Blood magnesium, Total bile acid, Aspartate aminotransferase, Gamma-glutamyl transpeptidase, Urea, Creatinine, Uric acid, T3, T4, TSH, CRP, FT3, FT4, PTH, Ca, Ultrasound description, Position, Size (mm), Echo properties, Form, Boundary, Edge, Echo distribution, Strong echo, Envelope contact, Blood flow, TI-RADS.
