# Supplementary material for: A machine learning-based approach to predicting the malignant and metastasis of thyroid cancer
Source: Front Oncol. 2022 Dec 19;12:938292. doi: 10.3389/fonc.2022.938292 (PMC9806162; doi:10.3389/fonc.2022.938292)
Supplement: Supplementary file 8 [file Table_3.docx]

Supplementary table 3. The hyper-parameter tuning range of different machine learning algorithms

| Machine learning algorithms | Hyper-parameter space |
| --- | --- |
| Logistic Regression | / |
| Ridge Regression | {‘alpha’: [0.001, 0.01, 0.1, 1, 10, 100, 1000], ‘solver’: ['svd', 'cholesky', 'lsqr', 'sparse_cg', 'sag', 'saga']} |
| XGBoost | {'learning_rate':[0.01, 0.015, 0.025, 0.05, 0.1, 0.2, 0.3], 'max_depth':[1, 2, 3, 5, 6, 7, 9, 12, 15, 17, 25], 'min_child_weight': [1, 3, 5, 7, 10], 'gamma':[ 0, 0.05 ,0.1,0.2, 0.3, 0.5, 0.7, 0.9, 1], 'subsample':[ 0.6, 0.7, 0.8, 0.9, 1], 'n_estimators': [50, 60, 70, 80, 90, 100]} |
